# Extracts and Gleanings

**Published:** 1872-01

**Authors:** 


### PART II.

### Abridged Extracts and Gleanings from our Exchanges

*MULTUM IN PARVO.*

---

On some Preparations of Iron.—We hold it to be something of a duty devolving upon every professional reader, however humble or otherwise, to contribute his mite towards the general stock of practical knowledge now being gathered in monthly for the pages of our Companion. I say *our* Companion, because every subscriber should feel that *his* professional interest is promoted in exact ratio to *its* success, as in every way identified with *its* welfare. We all have our favorite prescriptions—such as experience has stamped with the seal of reliability. From this field a vast fund of valuable information might be culled. In this connection I would passingly suggest, that we be careful to offer nothing for its pages which, when weighed in the balances of trial, will be found in the least wanting. In this spirit I briefly invite the attention of my brother co laborers to the subjoined preparations of iron, which we have been using constantly and with entire satisfaction for the last twenty-five years. Every experienced practitioner knows, that our best iron compounds are those having the *red oxide* as their base; and especially is *this* true, when our aim is the restoration of impoverished blood. *This* is the indication in quite a preponderance of the cases calling for iron. In *this very chemical, particularly*, consists the efficacy of the most mineral waters, as well as the advantages of the oxysulphates, over many others. Let me incidentally add, the first formula is no *therapeutical novelty*, but was invented and successfully used by Sylvester sixty years ago, and is said to have been in constant use ever since by many English practitioners:

OXYSULPHATE OF IRON.

| | | |
|---|---|---|
| R.—Chrystalized Sulphate Iron, | . | dr. ijss. |
| Nitric Acid, | . . . . | dr. iij. |
| Pure Water, | . . . . | oz. iss. |

Stir well the iron and acid, by constant rubbing in a glass mortar, for at least, 20 minutes—gradually add the water, and filter through white filtering paper. The result is a clear limpid fluid, which may be given in doses of from 5 to 10 or 15 drops, twice daily, in a little water or infusion of quassia—is easily prepared, and will be found, for general use, preferable to the muriated tincture of the shops—harmonizes chemically with, and confers solubility on quinine, sulph. magnesia, etc., and may be relied on as one of the very best restoratives for debility and torper of the liver, following successfully treated cases of hepatitis, or miasmatic fevers, in which the biliary organs have suffered. For chronic chills, the following formula, alone, will be worth four times the price of THE COMPANION to any practitioner engaged in a malarious district:

| | | | |
|---|---|---|---|
| R.—Sulph. Quinine, . . . | grs. | xxx. |
| Liq. Oxysulph. Iron, . . . | dr. | i. |
| Fowler's Sol., . . . . . | dr. | i. |
| Water, . . . . . . | oz. | ij.—M. |

S.—A tea-spoonful thrice daily, after meals, occasionally adding a small dose of sulph. magnesia to obviate costiveness. The iron and arsenic may be varied to meet the leading indication:

| | | |
|---|---|---|
| R.—Spts. Mindererus, . . . | oz. | ij. |
| Liq. Oxysulph. Iron, . . . | dr. | i.—M. |

This gives a beautiful florid mixture—palatable, and in doses of a tea-spoonful or so, thrice a day—not apt to disappoint as a general tonic. The ammonia promotes the action of the iron and imparts to it a decided *capillary and renal tendency* We have found the liquor oxysulph., in doses of from 3 to 6 minims with a drachm or two of the liquor ammonia acetat, every 3 or 4 hours, a valuable remedial agent in combatting the forming stages of tonsilitis, scarlatina, typhoid fever, and puerperal inflammations. In these affections, I am convinced of the efficacy of this combination as superior to the mur tinct. used in the same way.

SYRUP IODIDE IRON.

| | | |
|---|---|---|
| R.—Iod. Pot., . . . . | grs. | xxx. |
| Liq. Oxysulph. Iron, . . . | dr. | ij. |
| Simple Syrup, . . | oz. i. and dr. | ij.—M. |

Dissolve the iodide in the syrup and add the iron. This syrup is of a beautiful red color—will keep without deposition for years, and is a *most excellent tonic and alterative*, in doses of 20 to 60 drops twice a day. We will close by adding, if the brom. pot. or brom. ammonia be substituted for the iodide (correspondingly as to quantity) in the above formula, a rich golden colored compound will result and prove itself on trial, a remedy of the first class in all ænemic, chlorotic and nervous conditions resulting from uterine affections or otherwise.

GEO. D. HODGE. M. D.

*Holly Springs, Ark., Dec.* 11*th*, 1871.

CHLORAL HYDRATE IN INFANTILE CONVULSIONS.—I have found enemata of 10 to 30 grs. of Chloral Hydrate a specific for infantile convulsions, produced from any cause whatever, unless, perhaps, it be from some varieties of poisons where disgorging the stomach is an absolute necessity. It is well for the practitioner to know, accurately, how much he administers. This is the more necessary because the drug has not been on hand long enough for us to handle it with the same indifference as we do quinine and other old "stand-bys" of the profession. My custom is, to add the chloral to such quantity of water as will secure its dilution only so much as to prevent local irritation, but not enough for the bulk to excite peristaltic action of the bowels. Try it, and anxious parents and friends " will call you blessed," and you will leave your patient with a light heart. The quantity to be used in a given case, is to be determined by the age of the sufferer, ranging from, say, grs. v. to xl.

Midville, Ga. L. B. BOUCHELLE, M. D.

PUERPERAL CONVULSIONS SUCCESSFULLY TREATED WITHOUT BLEEDING.—It has been my fortune, in a practice of fifteen years, frequently to have this formidable malady to treat, and with the exception of *cupping* in the temples, in two cases, I have never found it necessary to resort to blood-letting. In the first case, I did it, as I now think, before I learned better; and, in the second, as a compromise between my own judgment and the established usages of the profession. My own remedies were not giving so satisfactory results as the urgency of the case seemed to require, and I never stand by and see a patient suffer and die, wi hout exhausting all the available resources in my knowledge. Instead of bleeding and cupping heroically, as the law is, I resort to brisk cathartics, and none act so promptly, in my hands, as oleum tiglii—croton oil—gtts. 1, 2 and 3, on the tongue, and encoiraged by warm salt-water enemata—Chloride of sodium, in aqua, as much as the water will dissolve. I have never yet met a case in which the convulsions did not yield promptly and permanently after the action of the remedy.

Severe cases have been, as is usual, followed by coma, and this state gives away immediately upon the action of a blister to the back of the neck. I would not have it believed, however, that I regard any remedy indicated before parturition, but delivery; but post-partum cases are alluded to exclusively, that do not yield after delivery. My success in these cases makes me strongly doubt the propriety of bleeding in any case, except in very rare instances. Certain it is that if the patient can be relieved without the loss of blood, the road to perfect health is much shorter, which is no mean consideration.

I only name chloroform to say, I never rely upon it, however willing I am to take it as an adjuvant. It does not, cannot, reach "the root of the matter." I hope the profession will give the croton oil, or anything else, a fair trial, that will insure to the patients relief, and conserve the vital energies of the system.

Midville, Ga. L. B. Bouchelle, M. D.

Internal Hemorrhoids.—Dr. Beekman has treated eleven cases of interal hemorrhoids, all occurring in females, and all treated without operation. In every case, the only internal medication consisted in the employment of the following formula:

R.—Pulv. Sennæ.
Potass Bitartrat.
Pulv. Sulphuris. . . . aa. oz. ii.
Pulv. Zinziberis, . . . oz. ss.—M.

This preparation is designated in the Dispensary Pharmacopœia, as *Pulvis Sennæ Compositus*. The dose as employed by Dr. Beekman, was a tea spoonful of the powder, in molasses, every morning. The local treatment consisted in the use of the following ointment:

R.—Ext. Belladonnæ,
Plumbi Acetatis, . . . aa. dr. ii.
Acid Tannic, . . . oz. ss.
Ung. Adipis, q. s. ut fiat unguentum.

A small mass of the ointment to be introduced within the anus thrice daily, after a thorough ablution of the parts with cold water. The duration of the treatment was quite various, bearing a direct ratio to the severity of the case, ranging from a week to about five weeks. As far as could be ascertained, recovery took place in every instance, and no case of relapse has thus far come to Dr. Beekman's notice. A few of these patients suffered from hæmorrhage, but not to an excessive amount. Instead of the ointment above mentioned, Dr. Beekman uses, in private practice suppositories made up of the same ingredients with the exception that cocoa-butter is substituted for the simple ointment—each suppository containing two grains, each, of extract of belladonna and acetate of lead, with four grains of tannin.—*N. Y. Med. Gazette.*

Kneading in Constipation.—By Geo. H. Savage, M. D., London. In Dr. Black's Lecture on Constipation (reported in the Journal, Jan. 28th), he alludes to "kneading" of the bowels as not being likely to do good. Having had one case in which such treatment—accidentally applied—saved the patient's life, I send a short account of it.

I was called to see a healthy old man, suffering from constiptation and excessive pain over the right hypochondrium, so severe as to hinder examination. Enemata were used; the pain lessened, when a tumor was felt where the pain had been; this was soft and semi-dull. Enemata were continued; sickness and vomiting followed, stercoracious matter being ejected. The patient seemed sinking; another medical man was called in consultation; and, there being a difference of opinion as to the nature of the tumor, it was manipulated several times. Soon after we left the house, the patient passed a large quantity of hard fæces, and found that the tumor had disappeared.—*St. Louis Med. and Sur. Journal.*

Improved Dover's Powder.—Dr. B. D. Keator, of Tolone, Ill., says the following formula is superior to the old Dover's Powder in every respect:

| | | |
|---|---|---|
| R.—Sulph. Morphia, | . . . | grs. x. |
| Pulv. Camph., | . . . . | dr. iii. |
| Pulv. Ipecacuan, | . . . | dr. i. |
| Creta Preparat., | . . . | dr. iii. |
| Pulv. Glycyrrh. | . . . | dr. iii. |

Thoroughly mix. Dose same as Dover's Powder, in water.—*New York Medical Gazette.*

Hooping Cough.—Dr. Bartlett's formula:

| | | |
|---|---|---|
| R—Argenti Ioduret, | . . . | gr. x, |
| Sacch. Alb., | . . . . | gr. lxx, |
| Pulv. G. Tragacanth, | . . | gr. viii or x.—M |

Rub well together, moisten with a few drops of water, make pill mass and divide into 80 pills. Each pill will contain one-eighth of a grain of the salt.

S.—To a child 2 or 3 years old, one pill 3 to 5 times a day. To children 6 to 10 years old, 2 pills at a dose. This, the Dr. says, is "safe, pleasant and effective."

Oxaluria.—Dr. H. S. Thorne, of Chicago, (Michigan University Medical Journal), has found the permanganate of potash of valure in oxaluria. He gives one grain either made into a pill or dissolved in water, on an empty stomach, three times a day.

AMENORRHŒA AND DYSMENORRHŒA.—Dr. E. Bradley, (Medical World) says: "Some time since, I became convinced that many cases of obstinate amenorrhœa and dysmenorrhœa depended upon a malarial poison, and I commenced the use of counter-irritation over the spine, followed by the local application of a saturated solulion of quinine. In quite a number of cases, during the past six months, I have used this treatment with marked success. The method of using the quinine is to first paint the spine over its lower two-thirds with the following:

R.—Oil Mustard, . . . . . dr. iij.
Alcohol, . . . . . oz. ij.—M., using a wide camel's-hair brush, and taking care not to allow the solution to cover a wider space than one or two inches. Then, when the surface is thoroughly red, apply the following:

R.—Quinine Sulph., . . . . . dr. j.

Aquæ, . . . . . . oz. j.

Acid. Sulph. Aromat., . . . q. s.—M.

In applying the essential oil of mustard, considerable pain is excited. This immediately ceases, however, on the application of the saturated solution of quinine.

In one case, in which suppression had existed for several months, the patient having had intermittent fever for some time previously, and having been treated by the internal administration of quinine *unsuccessfully*, after the use of the counter-irritation and quinine locally for about three months, the menses returned, and she felt perfectly well. In another case, there had been great neuralgia of the head and uterus, and it was speedily relieved by this treatment.

In another case of dysmenorrœa, with obstinate constipation of the bowels, the patient was entirely cured, and has not suffered from constipation since.

A NEW COUNTER-IRRITANT.—By Prof. Parker:

R.—Croton Oil, . . . . . oz. ij.
Spirits Camphor, . . . . oz. ij.
Alcohol, . . . . . . oz. vi.

SCARLATINA.—Dr. Sansom, gives 10 grain doses of sulpho-carbolate of sodium every 2 hours in Scarlatina, with happy results. He regards the sulpho-carbolate of iron as superior to the other preparations of iron, as a tonic.

INFANTILE WASTING.—Dr. Sansom esteems the sulpho-carbolate of calcium very highly in infantile wasting.

Collodion in Chilblians.—Dr. C. Green, in the Buffalo Medical Journal, says: "In Chilblains he has used, with the most decided success, Collodion. In one case the patient had her feet for time exposed to severe cold, which produced erythematious inflammation of several of the small toes. They were swollen, red, tender and itching. I completely inveloped them in a thick coating of collodion, and the contraction which took place on its drying, produced so much compression of the vessels, that the toes were as pallid as frozen ones. The pain and itching were relieved, and in twenty-four hours these members were nearly well. I have cured pernio of the heel also with this article.

Treatment of Psoriasis.—Dr. H. G. Pifford, (Med. Gazette) says: "The treatment of Psoriasis consisted chiefly in the use of Fowler's Solution, internally, and thorough applications of the tar ointment mentioned above, followed by the glycerate of tar or of cade as the condition improved. Several cases were treated with carbolic acid internally, but without encouraging result. Copaiva was prescribed for one case, but as the patient had previously used the same remedy for a different affection, he supposed that we had mistaken the nature of his complaint, and declined further treatment. Galvanism was employed in two cases.

Dysentery.—Dr. J. B. Brown, says that in the treatment of dysentery, he has usually been successful, in a few days, with the following formula:

| | | |
|---|---|---|
| R.—Ol. Amygdal. duli., | . . . | dr. iv to vij. |
| Gum Mimas, | . . . . | dr. ij to iij. |
| Aq. distillat, | , . . | oz. ij to iij. |

*Fiat lege artio emulsio ani adde.*

| | | |
|---|---|---|
| Sodæ Nitratis, Aquæ lauro cerasi | } aa. . . . | oz. ss. |
| Syr. Simp, | . . . . | oz. j. |

M. S.—A table-spoonful to be taken every one or two hours, after having been well shaken.

For children, I prescribe the half, or only one-third part of the quantity—and I found it an excellent means for overcoming, in their different forms, diarrhœa and dysentery. After the evacuations have become normal, the medicine is to be continued for one or two days, on account of great liability to relapse. In the more severe cases, I ordered the addition of a few grains of the extract of opium to the former mixture, in order to allay, more rapidly, the existing painful tenesmus and the nervous excitement in general.

PITYRIASIS VERSICOLA.—This disease, of the skin, is common; consisting of yellow spots, covering sometimes a large surface. It usually appears on the neck and shoulders, in flexures of arms, etc. Dr. G. Pifford recommends the following treatment:

"Our usual treatment is to apply a strong tinct. of iodine to *all* the affected spots, and to repeat the application morning and night until the epidermis begins to loosen. When this occurs, employ friction with a damp, coarse towel until all apparent trace of the affection is removed. After this the body should be bathed daily for about a week with sulphurous acid, and the patient charged to make frequent changes in his undergarment, and not to resume any which have been worn during the time that the affection was present without first subjecting them to thorough baking in an oven. If all of the spots have been removed by the iodine and friction, the patient may expect to be permanently cured; but if any of them, however small, escape notice, he must look out for a reappearance of the affection. Having once effected a lodgment, it will spread with great facility, but its transference to others is under the control of laws which are not as yet thoroughly understood.—*Medical Gazette.*

OXALATES IN THE URINE.—The following formula is highly esteemed by those who have used it:

| | |
|---|---|
| R—Acid Nitro-mur. dil., . . | f dr. jss, |
| Tr. Chirat., . . . . | f dr. iij, |
| Tr. Aconit., . . . . | min. xxx, |
| Infus. Aurant, . . . . | f oz. viij.—M. |

S.—A tablespoonful 3 times daily.

TREATMENT OF TYMPANY BY LOBELIA.—Dr. Bixby says: The employment of this drug for the purpose indicated, was novel to him, and from its general repute he should have been prejudiced against it. In the case referred to, however, where the tympany had been very marked, and had persisted for a couple of months, immediate relief was obtained by lobelia injections, in the proportion of one dr chm of the tincture to an ounce of water. Dr. B. had never seen so sudden and so satisfactory a result produced by any remedy, as in the instance referred to. There had been little or no constitutional effect.

Dr. Bixby related a case of violent and prolonged epistaxis checked by a current of salt-water passed continuously through the nares by a fountain syringe, for the space of two minutes.—*Jour. of the Gynœcological Society of Boston.*

Sympathetic Nervous Trouble from the Presence of a Tenia.—Two cases, mentioned by Dr. Maurin, (in Sud Medical No. 5, 1870, and Lyon Medical, April 24, 1870,) are interesting in this, that they might have given rise to a diagnosis of troubles of the nervous centres.

The first patient was a clerk, aged forty-two, who, for fourteen months, had experienced more and more serious illness. At first he suffered from dull pains in the epigastrium; these pains, after a while, took on the character of an intense gastralgia with inexplicable remissions and exacerbations.

A few months afterward he had some vertigo complicating the gastric pain. Finally, for eighteen months past, the gastralgia has yeilded, but has been followed by a continued sensation of vertigo, a fixed pain in the nape of the neck and between the shoulders; sensation of falling forward when the patient walks up or down any incline; sensation of a soft body, like a cushion under the feet during the walk; fear of an imminent death; sudden inclination to commit suicide.

The second patient was a physician who experienced all the symptoms of a cerebral congestion; cephalgia, troubles of the intellect, incomplete amnesia, impediment of speech; the patient being gouty had fear of a metastasis; but a few articulations of tenia having been passed, the diagnosis was made, and, as in the preceding case, a dose of kousso, by producing the expulsion of a tenia, caused at the same time all his symptoms to disappear.—*New York Medical Journal.*

For Corneitis.—The following formula (Dr. Geo. Lawson's) has given excellent results.

| | |
|---|---|
| R—Hydrarg Protoiodid, . . . | gr. ss, |
| Atropiæ Sulphatis, . . . . | gr. ij, |
| Plasmæ Amyli, . . . . | dr. iv.—M. |

S.—Apply with probe to lower lid once a day.

The compressive bandage should be used in all forms of corneal inflammation.

Frozen Beef-Essence.—Dr. H. B. Hare, states (Phila. Med. Times, Oct. 16, 1871,) that in a case of scarlet fever in a child, the patient could not be induced to swallow the beef-tea which his condition required. As he took ice with avidity, the father suggested that if the beef-tea were frozen he might then be induced to take it in that form. The suggestion was carried out, and the child took the frozen beef-tea readily. This expedient may in many cases be advantageously resorted to.

Bromide of Sodium.—Dr. Meredith Clymer, (Medical World) remarks: "For some time past, I have habitually used the bromide of sodium in all disorders of the nervous system where before I prescribed the bromide of potassium, and, so far as my own experience goes, speak positively to this point. I have given it in a number of cases of epilepsy continuously for months without any of the unpleasant symptoms which so constantly follow the prolonged administration of the potassium salt, excepting the eruption, and with the best results in mitigating or suspending the paroxysms. Dr. Decaisne has given the bromide of sodium for a year without its producing the systemic saturation so frequent during the long and continuous exhibition of the bromide of potassium.

The taste of the bromide of sodium is much less unpleasant than that of the bromide of potassium, being very like common salt, and it may be used to replace the latter, mixed with the food, as with bread and butter, eggs, in milk, etc. Hence it is of more easy administration than the bromide of potassium, to the taste of which some persons have invincible repugnance, and increasing with its use.

The doses of bromide of sodium are about the same as those of bromide of potassium. In epilepsy, I usually give 20 grains three times daily, and have rarely gone above that amount. It sometimes seems to cause or encourage constipation.

Skin Diseases.—When burning pain is complained of the following will be found to be excellent:

| | |
|---|---|
| R—Starch, . . . . | dr. vi, |
| Ox. Zinc, . . . . | dr. iij, |
| Cochineal, . . . . | gr. j, |
| Powdered Camphor, . . | dr. ss. |
| | Mix thoroughly. |

Or the following soothing ointment:

| | |
|---|---|
| R—Subnit. Bismuth, . . . | dr. ss, |
| Rect. Spt., . . . . | dr. ss, |
| Simple Ointment, . . . | dr. vj, |
| Oil of Roses, . . . | min. ss,—M. |

Or the following lotion:

| | |
|---|---|
| R—Sol. Subacetat Lead, . . . | dr. i, |
| Glycerine, . . . . . . | dr. iv, |
| Distilled Water, . . . . | oz. vi.—M. |

Turpentine in Ring-worm.—Von Erlach and Lucke recommend Spts. Turpentine brushed lightly over the surface, as a remedy in common ring-worm.

Deterioration of Milk in Feeding-Bottles.—Prof. Gunning (Medical Times and Gazette, May 5, 1871), Amsterdam, writes, "I object to the infants' feeding bottles in all instances where any part of them is composed of caoutchouc or india-rubber, or any like material. There is nothing so ill suited to the constitution of the human body as the material in question. When, in consequence of suction, the pores of the caoutchouc are enlarged, some portion of the milk always remains behind in them, which cannot, or at least cannot without great difficulty, be removed. This milk quickly becomes bad, and spoils the fresh milk with which it comes in contact. The caoutchouc material in question is made up of several ingredients. White zinc, or white lead, is very commonly employed, which is very poisonous. My objections are not founded exclusively upon *a priori* conclusions. In this country many fatal cases have happened among infants, which on solid grounds may be ascribed to the use of these bottles."—*Cincinnati Med. Rep.*

Therapeutical Incompatibility of Quinine and Veratrum Viride.—Dr. Bradley, of St. Mary's, Ohio, reports that when a patient is under the influence of Verat. V., it is highly dangerous to administer quinine. The effects are alarming; immediate sinking of the pulse, which in some cases reaches collapse.—*Cincinnati Lancet and Observer.*

The Sulpho-Carbolates as Gynæcological Agents.—Dr. Mack had found a special advantage from the sulpho-carbolate of zinc. In cases of ulceration or erosion of the os and cervix, he had employed it by irrigation, in the proportion of half a drachm of the salt to the pint of tepid water. As an intra uterine application, he had applied it both by injection and with a mop, and had found it to relieve septicæmia, whether used before or after the removal of decomposing matters from within the cavity of the uterus. It was also beneficial in chronic vaginitis, attended with fetid discharge; being used in solution with glycerine, in doses of ten grains at a time, applied by the tampon.—*Jour. Gynæcological Society of Boston—Proceedings of.*

Nauseous Medicines.—Dr. Bixby suggests, with reference to the administration of food or nauseous medicines to patients whose stomach was unusually irritable, as in so many gynæcological cases, that it was often of use to precede them by the administration of a single peppermint. He had first noticed the effect by an experiment upon himself. He had found that he could thus smoke tobacco for a longer period without being nauseated.—*Ibid.*

Hypertrophy of Os Uteri Treated by Puncture.—Dr. John Scott, says: "I at first used a blade which was much too narrow to enable me to deplete the organ effectually, but I afterward changed it for the one which I now show you, and which resembles a bleeding-lancet, but is only half the breadth, has a well-tempered point, and a fine-cutting edge. I am fully aware that in puncturing the cervix with a lancet, for the purpose of reducing engorgement by local depletion, I can lay no claim to originality, for it has been done by several before me; but what I do lay claim to is the carrying out this treatment persistently and consecutively, and with results for which I was totally unprepared by previous reading or experience, and which I have since verified to a degree which leaves on my mind no doubt whatever, of the exceeding value of the treatment. From the first the benefit has been most marked, both locally and generally, and my two colleagues in the Woman's Hospital can bear testimony to the truth of my statements. The diminution of engorgement, the absorption of hypertrophied tissue, and the healing of erosions, with relief to local pains and the improvement of the general health, are so marked as to be indubitable; and if steadily and judiciously persisted in, complete and perfect recovery is the almost certain result. But the greatest triumph which the treatment has achieved in my hands has been the removal of flexions which had hitherto baffled all my efforts, and with them the absorption of adhesions which had existed for years, and which had firmly tied down the uterus in its false position. The depth of the puncture is a matter of the highest importance; for if too deep it will not only cause pain, but will absolutely increase the mischief it is intended to remedy; while if too shallow, the mucous membrane and capillaries only are divided, and the beneficial effects are but transitory. One-sixth of an inch is the utmost extent to which I penetrate, and in cases of flexion, I chiefly direct my attention to the posterior or anterior lip of the cervix, as it happens to be a retroversion or an anteversion. Having punctured, I apply, by the sponge-holder, warm sponges, to increase the bleeding, and if the latter should continue longer than is desired, it can be easily controlled by a little prolonged pressure of the sponge to the bleeding point, subsequently brushing it with a little solution of perchloride of iron and glycerine. The punctures are made every four or five days, as the state of the organ may demand, beginning about the third day after the cessation of the catamenia, and ending about three or four days before its commencement. I do not mean to say that puncture constitutes the sole treatment in all the cases I have alluded to, but it constitutes the main treatment, and for its entire safety and its great efficiency, I can honestly vouch.',

DYSPEPSIA—CONSTIPATION.—Dr. T. Curtis Smith, (Cincinnati Lancet and Observer,) regards Nitro-Muriatic Acid of great value in these diseases. He says: "I have been in the habit of preparing or prescribing the following solution: R.—Acid, Nitro-muriatic, dr. i.; Aque Puræ, oz. iv., or instead of water, simple syrup or cinnamon water. Of this, a tea-spoonful, just before commencing to eat, in a half glass of water. If taken even a few minutes before eating, or after it, the mouth should be rinsed with a weak alkaline solution, generally a weak solution of bicarb. soda, which article is nearly always immediately at hand, for baking purposes, and can be prepared *ad libitum.*

In most cases it is best to precede the use of this remedy by a mild cathartic, as the pil. rhei. comp., or a few grains of colocynth comp. ext., with an aromatic. Where there is a heavv yellow coating on the tongue, the pil. cath. comp. is best (of course barring acidity, which should always be removed before the administration of mercurials); but where these are used, the acid should be deferred one or two days to prevent ptyalism, which is always an unpleasant complication.

In addition to the diseases above mentioned for which this remedy is recommended. there are many cutaneous affections in which, as an alterative, it is highly beneficial.

GLEET.—Dr. A. R. Finck, treats this disease as follows: "T. McL., had gleet for four years, which resisted the prescriptions of many physicians. I anointed a medium sized bougie with ung. hyd. mit., (lard and mercurial ointment—half and half,) and pushed it down his urethra to the bulb, It was left *in situ* eight or ten minutes, and rotated about a-half dozen times to rid it of the ointment, and *stir up* the old sore. A moderate inflammation and increased flow was developed, which was treated by rest and a saline laxative only, and the complaint disappeared. This treatment was instituted early in the summer. The patient was a journeyman bricklayer, who left my neighborhood when the season's work was over, up to which time he had no more "show."

IODINE IN VOMITING.—Dr. Caspari, of Horn, has for the last twenty-five years successfully prescribed the tincture of iodine in cases of obstinate vomiting, where nothing else relieved the patient.

IODIDE OF STARCH POULTICE, FOR SLOUGHING SORES, ETC.—Take two ounces of starch and mix with six ounces of boiling water to form a jelly, then add, before cold, half an ounce of tincture of iodine. The poultice is now ready for use.

Infantile Remittent Fever.—Dr. Wilks said the whole subject of the so-called "infantile remittent of children" required reinvestigation; it was clear that much of the disease was typhoid, and then, of course, the ordinary treatment was adopted; when the disease was due to stomach or bowel disturbance, and was more distinctly what had been styled gastric fever, then medicines to improve the condition of the alimentary canal were necessary, and for this purpose the various aperient powders in the *Pharmacopœia* were very useful. At the Royal Infirmary for Children, where a routine practice is necessary, Dr. Wilks gave the pulvis rhei salinus (rhubarb and sulphate of potash) once or twice a day, for a few doses, and then some quinine. This compound was invented by Dr. Fordyce, physician to St. Thomas' Hospital, who himself thus spoke of its merits: "Had I been more ambitious of dying a rich man, than of living a useful member of society, the powers of our anti-hectic powder of curing, as if by miracle, the hectic fever and the swelled bellies of children in this town, would have remained a secret while I lived."

Uterine Catarrh.—Dr. T. Parvin, Louisville, Ky., uses the following formula:

R—Carbolic Acid, . . . . dr. vi.
Glycerine and water, aa . . dr. i —M.

Applications may be repeated in from four to seven days.

Tincture of Veratrum in Pneumonia of Children.—Dr. Jacobi, in a clinical lecture before the New York Medical Journal Association, says: In the acute pneumonia of a baby, I would give a drop of the tincture every hour; to a child four or five years old, perhaps two drops every hour. If the attendant is intelligent enough to count the pulse, I say bring down this pulse to one hundred and ten or one hundred, but not lower; because when the pulse falls lower, the drug is apt to cause vomiting and temporary collapse. To obviate local irritation of the pharyngeal or gastric mucous membranes, I give the tincture in barley-water. This drug has no cumulative effect like that of digitalis. It will bear combination with quinine, and I think this is an important point. I often combine quinine with veratrum or digitalis where I want to get, not a speedy, but a continued effect upon the pulse, especially in the pneumonia of a debilitated child, where you are in doubt about stimulating to any great extent; where you do not know whether you ought to commence with benzoic acid, camphor, etc., you will control the pulse better with quinine and veratrum than with the latter alone. I ought to add that, in most cases, it is advisable to combine opium or hyosciamus with veratrum to obviate local gastric irritation.—*New York Med. Record.*

Malignant Small-Pox Treated with Sulpur Fumigations, and Sulphurous Acid Internally.—F. Hjaltelin, M. D., Chief Physician of I eland, Rekjavik, says: Every summer, and even ear.y in the spring, Iceland is generally surrounded by many hundred French fishing-vessels, which, to tell the truth, are never welcomed by the Icelanders; this spring, these vesseles were still more dreaded than ever, because accounts had been received of a malignant small-pox, raging over the unhappy French country.

In the beginning of April last, several French vessels came into the harbor of Rekjavik, and in some of these a third part of the crew had been seized by the small-pox, and some had died. A quarantine was at once erected in the neighborhood of our town, and as the chief physician of this island, I had to treat the sick sailors, and see that the quarantine regulations were strictly followed. Many of the patients brought into the quarantine had the confluent small pox, and were in great danger. I treated them all in the same manner, viz: on the plan of disinfection, advocated by me a long time ago. Sulphur fumigations were used in the sick room, and a mixture of sulphurous acid in water was given internally. The result has been most satisfactory, and I shall give a full account of the cases treated, in Dr. Dobell's Reports this year. As no cases of small-pox have, after the lapse of thirty days, occurred among the Icelandic population, either in the town or its neighborhood, I entertain the hope that the disease may be looked upon as stamped out by the aforesaid precautions. The people have been vaccinated and re-vaccinated, as a matter of course, and this may serve to protect the Icelandic population from this dreadful malady.—*Boston Medical and Surgical Journal.*

Treatment of Capillary Bronchitis in Chiddren by Warm Vapor.—Prof. Abelin, in the earlier period of practice, adopted antiphlogistic treatment, and rarnly saw a child recever. Subsequently he prescribed tonics and stimulants (quinine, musk, camphor and turpentine), with better results; but all these remedies were far surpassed in value by the mode of treatment long employed in his hospital, by the respiration of warm vapor, or rather of placing the patient in a hot-air bath. The children were placed in a properly constructed small chamber, in which was a vessel of water that was kept boiling day and night. Here the patient was retained for days and even for weeks, until complete recovery, which, however, usually soon took place. The result of comparison with other modes of treatment was most satisfactory. The percentage of death, which in 1864, amounted to 48, diminished in 1868 to 18. M. Abelin has also found great benefit from breathing the vapor of hot water in pneumonia. Lobular pneumonia may likewise be thus treated, and here, in addition, turpentine embrocations and cataplasms are to be supplied. In lobular pneumonia M. Abelin first gives calomel, or if diarrhœa be present, small doses of calomel with opium or morphia, inf. ipecacuanhæ, with vin. livuiritiæ, thebaicum, and syrup-scillæ, and as soon as the symptoms give way a turpentine emulsion internally and fly blisters externally.—*Journal fur Kinderkrankheiten.*

UNWHOLESOME CANDIES.—Hermann Endemann, Ph. D., Assistant Chemist to the Health Department of New York, has recently examined a great variety of candies, collected from various dealers by Drs. Leo and Prankell, Assistant Sanitary Inspectors:

INORGANIC ADULTERATIONS were detected in only two cases, in both cases in lozenges, to the extent of 3 and 6 per cent. In one establishment visited a white powder was obtained, which proved to be gypsum, sulphate of lime.

COLORING MATTERS.—Reds were either carmine or aniline red, both harmless.

Blues were either ultermarine or Prussian blue, both harmless.

Yellows were either saffron, chromate of lime, chromate of baryta, chromate of lead, gamboge, or yellow vegetable colors precipitated by alum and chalk.

Of ten samples examined, five were colored with chromate of lead, and one with gamboge, both of which are poisonous.

Greens were found to be harmless.

FLAVORS.—Oil of peppermint is often adulterated with oil of turpentine. The other flavors are generally artificial ethers, as, for example, butyric ether. Many of these are considered injurious.

SUBSTITUTES FOR SUGAR AND GUM-ARABIC —Glucose, starch-sugar, is common in some kinds of candy. Starch is extensively used as a substitute for the more expensive gum-arabic. Both of these substances are harmless.

In conclusion, the public is cautioned against highly colored yellow, orange and green candies, and against highly-flavored candies.—*American Chemist.*

ULCERATED SORE-THROAT.—Dr. Sansom uses the sulpho-carbolate of Sodium with great success, rapidly curing his patients. He gives half drachm doses.

Method of Promptly Relieving Facial and Dental Neuralgia.—This method consists in turning into the meatus auditorius from four to ten drops (according to the age and sensibility of the patient) of the following fluid; then to close the opening of the ear by means of a little cotton, and to cause the patient to hold the head inclined for some minutes to the side opposite to the seat of the pain so that the liquid may remain in the bottom of the ear. This preparation is thus made:

R.—Ext. Opii,

Ext. Belladonna.

Ext. Stramonia, } aa., . parts j.

Aq. Pruni Virg. . . . parts xij.

Solve et cola.

Although this preparation may be only extemporaneous, it may nevertheless, be preserved, if care is taken to keep it cool, by pouring on its surface from two to four drops of sweet almond oil.

It is very rare, with the use of this liquid, that relief is not obtained in a few minutes, and the patient asleep in half an hour, whatever may have been the severity of the pains, and that without having been in the least danger. Absortion takes place almost as rapidly as from a denuded surface, and it is, therefore unnecessary to blister the patient when we wish to use narcotics, since they act almost as rapidly by the auditory passage.

If it should happen that at the end of eight or ten minutes, the pain does not yield to the remedy (which sometimes happens when the quantity used has been too small, or when we have to treat a neuralgia which has already required the use of narcotics in any way) it is necessary to use a second dose, at least equal to the first, but in the opposite case, in order to obtain promptly, that relief which is only too frequently momentary of facial neuralgias of long standing.

Aconite to Children.—Dr. W. M. Chamberlain, (Medical Record), regards the tincture of aconite a valuable cardiac, and arterial sedative, also a diaphoretic, in the febrile complaints of children; he gives one-half and one drop doses of the officinal tincture, largely diluted in water, and repeated once in two or three hours. The views of Jacobi upon camphor, quinine, and ergot, that they act as sedatives by their effect upon the vaso-motor nerves, has interested him; but the effect of the latter agent he considers analogous to sulphuric acid, which has quite a reputation as an anti-febrile remedy. He is not aware that it either reduces the power or frequency of the pulse, or lowers the temperature of the body.

To Expell Foreign Bodies from the Larynx.—Dr. Hansford, of Illinois, says he has expelled watermelon seeds, a grain of corn, a glass bead, a pin, from the larynx, by directing the patient to lie upon a bench, face downward, with head projecting over the edge, and to take a deep inspiration. He then, while the lungs are filled with air, gives a smart blow between the shoulders with a pillow made hard by compression.

Treatment of Hemorrhage from Teeth.—Dr. W. H. Waite, in the Canada Journal of Dental Science, recommends strongly, in cases of troublesome hemorrhage following extraction of teeth, the use of a saturated solution of alum. He packs the empty alveoli with a compress of cotton saturated with the alum solution.

Alterative and Antispasmodic Mixture (Tanner's,).—Used in third stage of croup, in some cases of acute bronchitis and in some forms of pneumonia in children:

| | |
|---|---|
| R.—Potass. Iodidi, - - - - | gr. viij. |
| Tr. Hyosciami, - - - | gtt. xij. |
| Tr. Assafœtidæ, - - - | f. dr. j. to iij |
| Decoc. Senegæ, - - | ad f. oz. iss.—M. |

S.—Tea-spoonful a dose to a child one or two years old.

Hooping-Cough.—Dr. Curry, (Baltimore Medical Journal,) says: "The rational treatment that I adopt in this disease is outdoor exercise in good weather, mineral tonics, sponging the chest daily with cold water, easily digested food, attention to the secretory system, avoidance of all mental emotions, and chloral hydrate to allay nervous irritability, or, if the practitioner prefer, let him employ the bromides, belladonna or hydrocyanic acid. In the third or declining stage I give tonics and guard against sudden changes of the atmosphere. The natural tendency in this disease is to recovery. It is only those cases that run into one of its complications that swell the mortality list.

Chloral to Infants.—Dr. H. P. Farnham, (Medical Record) says: As a hypnotic, he has given the hydrate of chloral in one and a half grain doses to children six months old. He stated that although more palateable when given in combination with a syrup, yet it was prone to decomposition, to prevent which he usually added a few drops of chloroform to the mixture.

Diptheria.—Dr. Sansom gives 10 grain doses of sulpho-carbolate of sodium every 4 hours, with satisfactory results, in diptheria.

Bloody Discharges After Micturition.—Dr. Beltzell (Baltimore Medical Journal) says that he has used the Bethesda water in cases of kidney diseases very satisfactorily, that he tried it in these cases with dr. i. fluid extract matico to a pint of the water, and had gratifying results. He was at a loss to know the cause of these cases, for the urine, although excessive, was normal in its consistence. The books did not satify him on the subject.

Hooping-Cough Aborted.--Dr. Beltzell (Bal. Med. Jour.) says, that he has found that vaccination often appeared to abort pertussis.

Diebetes.—Dr. J. Y. Shearer reports (Med. and Sur. Reporter) a case of diabetes which he treated as follows:

R.—Tannic Acid, . . . . . grs. x.
Opii Pulv., . . . . . . gr. ss.

Three times per day—tinct. ergot dr. i., before each meal. By a week, under this treatment, the urine voided at night was reduced to one gallon. The next week, the tannic acid was increased by five grains, the opium by half a grain, and the tinct. ergot by one drachm. During the next week, the quantity of urine was reduced one-half. Again the tannic acid was increased five grains, and the tinct. ergot one drachm, the opium remaining the same. Condition of patient was much improved. Next week the tannic acid was increased to twenty-five grains, the opium and ergot remaining as before. Though the patient progressed favorably for the next week, the doctor thought he had not obtained the full effect of the medicine, so he increased the tannic acid to thirty grains, opium remaining the same, and tinct. ergot four drachms. This treatment continued for a month in full doses, and for a month in diminished doses, left the patient perfectly restored.

Rheumatic Ophthalmia (Dr.—Power's Formula):

R.—Tr. Aconiti rad.. . . . min. xxiv.
Tr. Colchici, . . . f. dr. ijss.
Aquæ Camphor, . . . f. oz. iv.—M.

S.—A table-spoonful 3 or 4 times a day.

Irritability of Stomach.—Dr. Hartman related (Baltimore Medical Journal,) a case of Bright's disease, associated with great irritability of stomach, which acknowled no control until the whey of cream of tartar was used. This remedy was retained without difficulty, and six hours after its administration oz. viii. of urine were passed, and 24 hours ½ gallon. In ten days he was discharged well, and has continued so since.

TREATMENT OF CHOLERA INFANTUM.—Dr. Hubbard in the Medical and Surgical Reporter, highly recommends sub-nitrate of bismuth and tannic acid in the treatment of cholera infantum. He says that under the old treatment detailed in the books, he lost most of his cases, but with the above, and paregoric to allay pain, he has not lost one of out fifty. The dose was varied to meet particular indications, but the general plan was the same in all the cases.—*Detroit Review of Medicine.*

COD-LIVER OIL AND ARSENIC.

| | | |
|---|---|---|
| R.—Olei Jcoris Aselli, | - - - | f. oz. ii |
| Vitelli Ovi, | - - - - - | No. i |
| Liq. Arsenicalis, | - - - | Min. xliv |
| Syrupi, | - - - - | f. dr. ii |
| Aquæ Font., | - - - | q. s. ad f. cz. iv |

S.—Tea-spoonful 3 times a day, after meals.—*Erasmus Wilson.*

APHASIA.—Dr. James H. Curry relates (Balt. Med. Jour.) a case of "aphasia," in which there was perversion of language, inability to speak correctly, and stammering. There had been neither apoplexy or paralysis—no apparent lesion. He gave half dr. doses bromide potassium every fourth hour. Next morning she had control of her voice. In the evening, when he visited her, she seemed forgetful of the occurrences of the day—forgot that he had seen her in the morning. She had had a slight pain on the left of the head previous to this affliction coming on. On the third day she was to all appearance cured.

*Cold Water as an Oxytocic.*—Dr. S. H. Garvin, (Am. Jour. of the Med. Sciences,) esteems cold water one of our most efficient remedies, when externally applied, in inducing contractility of the uterus. He says:

"After having made water thoroughly cold by putting sufficient ice in it, a cloth (nothing better than a large coarse towel) should be dipped in it, and then wrung or squeezed between the hands, until only sufficient water remains to wet the part to which it is to be applied, without running down around the patient; this should be quickly placed upon the abdomen, so that as much of the cold will be retained as possible; the cloth should be changed just as soon as it begins to become warm, from ten to fifteen minutes being generally sufficiently long to restore the pains to natural power and frequency. Thus applied, cold water, I believe, will excite, by reflex action, the uterus to contraction in any case, providing the nerve force be not so far expended as to fail to respond to the excitation of a uterine motor stimulant.

Inunction in Diseases of Children.—Dr. W. M. Chamberlain (Medical Record) speaks of Inunction as being a very old remedy, and pretty generally approved by the profession; he generally used fresh lard. The *modus operandi* of this form of treatment was not very apparent to him; the only rational solution seemed to be the protection that it afforded to the cutaneous nerves from the air. Opium he seldom uses in the febrile state of children, except occasionally in the form of Dover's powders. In peritonitis, pleuritis, or pericarditis, where the leading indications were to restrain motion of the serous surfaces upon each other; also in dysentery and muco-enteritis, its use has been followed with the best results. As an anodyne in the fever dependent upon local pain, and as an anti-spasmodic to the non-straited muscles, no remedy could compare with it. But its use in children is hazardous, and it should be prescribed with great care.

For Infantile Eczema.

R.—Vini Ferri,
Syrup Tolutani, - - - aa. f. oz. ss
Liq. Arsenicalis, - - - min. xii
Aquæ Anethi, - - . f. oz. i.—M

S.—Tea-spoonful to child 2 years old, 3 times a day, after meals. This is almost a specific.—*Erasmus Wilson.*

Hot Water in Hemorrhage.—Dr. C. Prince spoke, (Medical Record), of the effect of hot water in arresting hemorrhage, and mentioned a case of cancer of the uterus, from which the hemorrhage was very troublesome. Astringents and sedatives were used without benefit. The patient fell into the hands of another medical man, who applied hot water locally, with the effect of promptly checking the hemorrhage.

Cases of Palpitation of the Heart.—Dr. Warren Stone, of New Orleans, (Medical Record), reports two cases thus:

I was called to a gentleman, five years ago, who was in an extreme condition. He was dyspeptic and subject to palpitation. He said he had spent Sunday out of town with some friends, ate rather freely, and was afterwards subjected to severe excitement, which brought on agitation of the heart, and he was barely able to reach home. When I saw him his heart was acting with great irregularity, the circulation very much obstructed, and grew worse in spite of all I did. He became cyanosed, his urine was suppressed, and he expectorated specks of blood. He was ordered an ounce of the tincture of digitalis, with half a drachm of saturated tincture of veratrum, to be taken in doses of a teaspoonful. The effect of this was watched, and in a little more than twenty-four hours, he took the whole, the heart was acting naturally, and all the functions were established. On careful examination no organic disease could be detected. I do not give so large doses usually, but the case was extreme, and the engorged state of the organs did not admit of ready or free absorption, and the case being under constant observation, the medicine was continued, as no ill effect was produced when the circulation was established. This gentleman is alive yet, and is now traveling in Europe, and as I have heard, with great benefit to his general health.

Mr. H., a stout and rather plethoric man of sixty-six, a pretty free liver, sent for me in the fall. I found him laboring under the symptoms of heart disease in the last stage. He was sitting in a chair, resting his head on a pillow placed on a table, his lower extremities were very much swollen, and he had some water in his abdomen. The heart was extremely irregular, but no murmur could be detected. His urine was scant and high colored. I ordered him small doses of calomel, and the tinct. of digitalis and veratrum, in doses of twelve minims of digitalis to three of veratrum, three or four times a day. This quieted the heart very much, and the calomel soon began to increase the flow of urine, when it was assisted by the camp. powder of jalap, as often as his condition would bear. Under this course the dropsy disappeared, the heart became pretty regular and soon gained his strength and felt as well as he had for some years. He told me that he had been subject to the irregularity of the heart for many years, but he was a vigorous man; and although he occasionally had temporary dyspepsia, his food was appropriated well, and the muscles of his heart did not degenerate, and dilatation was scarcely perceptible. The next year he was elected to Congress, and as his politics did not please the party in power, his seat was contested. He spent an exciting winter in Washington, and he returned in a worse condition than he was before. I thought he must die, but I put him on the same course of treatment, with a little addition to the quantity of digitalis and veratrum, and he recovered slowly. I kept him under observation for some time, regulated his diet, exerciser etc., and kept him under the use of the digitalis and veratrum for some time. He went into business, and was able to exercise freely. I had not seen him for a year or more, when he called on me to know whether he might not resume his medicine again, as his heart began to be unruly, as he termed it.

Epithelima Removed by Bromine.—Dr. Wynn Williams showed to the Obstetrical Society of London, " a patient, nearly the whole of whose lower lip had been removed for epithelioma eighteen months previously. The disease shortly appearing in the cicatrix, the growth was successfully treated by two injections of bromine, twenty drops to a drachm of spirit. There was no appearance of disease at present."—*Lancet.*

A Case of Calculus Nephritis.—Dr. Wm. M. Champers, of Effingham, Illinois, (Chicago Medical Examiner), reported a case of calculus nephritis, in which recovery followed the subjoined treatment:

Externally, a blister; internally, with the following mixture:

| | |
|---|---|
| R—Potass. Acetatis, . . . | oz. ss, |
| Vini Colchici, . . . . | oz. ss, |
| Spts. nitrosi dulcis, . . . | oz. ij, |
| Tinct. Opii. Camph., . . . | oz. ss, |
| Fl. Ex. Belladonna, . . . | dr. j, |
| Aq. Cinnamoni, . . . . | oz. ss.—M. |

Sig.—One teaspoonful every three hours.

A calculus of phosphate of lime, weighing five grains, passed the urethra the day following. .

*Essence of Turpentine in Parasitic Diseases of the Head.*—Dr. Erlach, of Berne, remarks that in order to destroy parasites, which are the cause of several diseases of the hairy scalp, Kiichenmeister recommended the application of alcohol, which retards the development of spores and mushrooms. Experience has shown, however, that the action of alcohol does not extend to the fungi that are found in the hair follicles. Tincture of iodine acts better than alcohol; nevertheless the treatment by this remedy does not last more than three months, even in the most favorable cases. He has found, however, that the application of the essence of turpentine by means of a brush is more certain and rapid than all other methods of treatment. He believes he has thus cured a case of herpes tonsurans in seven weeks; several cases of mentagra in a week.—*Practitioner, from Giorn. Ital. delle Mal. Venere.*

Puerperal Convulsions.—Dr. L. G. Armstrong (Northwestern Medical and Surgical Journal), in a valuable essay read before the State Medical Society of Wisconsin, says:

"I wish to add my mite to the praise of chloroform in cases *where the woman is anæmic*, and where the case had been thoroughly bled, and yet the symptoms of returning convulsions, kept up in my mind an uneasiness, fearing they would return. In such cases chloroform cannot be used amiss, and is the remedy *par excellence.*

*The Use of Earth as a Dressing in Severe Burns.*—In severe burns of all degrees, Dr. Addinell Hewson (Medical Times), advises the use of earth, applied in the form of a dry powder or thick paste. He applies earth powder or paste to the affected surface; over this he places waxed blue paper, and confines all by the bandage. It is claimed that by this method the pain is greatly relieved, the discharge absorbed, fœtor diminished, and that the healing process is quickened. Reparation by scabbing is promoted, and the production of nodular cicatricial tissues prevented. —*American Practitioner.*

Aperient and Alterative.—Useful in children suffering from dispepsia, with offensive breath, acid eructations, sour evacuations and constipations:

R.—Sodæ Bicarbonitis, - - - grs. xx
Tr. Rhei, - - - - f. drs. ij
Infus. Columbæ,
Decoc. Taraxaci, - - aa. f. dr. vii.—M

S.—Two tea-spoonfuls to be taken night and morning. For a child one year old.

The Use of Acetic Acid in Affections of the Conjunctiva and Cornea—By Dr. B. A. Pope, of New Orleans.—Dr. Pope employed acetic acid of specific gravity, 1,041 (No. 8), which he says is a mild escharotic when of this strength. With it he treated a rebellious case of warty degeneration of the palpebral conjunctiva, for which ordinary caustics and excision had very slowly effected a cure, but could not preserve against relapse. The second attack was cured by the acid in less time, and finally, it was applied by a very fine camel's-hair brush once every day, and only to the spots to be destroyed. Other cases thus treated were the relaxed and hypertrophied state of the conjunctiva in the *cul-de-sac* following chronic conjunctivitis—some cases of trachoma in the stage of development, as an occasional application, and strictly confined to the granulations—an inflamed pinguecula, which the patient refused to have excised—hypertrophy of the caruncle and semilumar fold in pterygium—in two cases of calcareous degeneration of the epithelial layer of the cornea sometimes combined with excision—in a case of dense opacity of the cornea, the result of partial sloughing after ophthalmia neonatorum. When put upon the cornea, the acid will cause an ulcer after two or three applications, and care must be taken not to let this process make unmanageable progress—with such vigilance the new tissue which repairs the ulcer was found to have a grati fying degree of transparency. It needs to be repeated a number of times to attain the best result, and is a remedy which only a skillful hand should apply, and an experienced eye watch, but doubtless it may do good service in some intractable cases, as enumerated.

TREATMENT OF PUERPERAL ECLAMPSIA.—Dr. Henry Jewett, Canandaigua, New York, (Buffalo Medical and Surgical Journal), says:

If puerperal eclampsia is the result of uræmia, or any other poison acting upon the excitomotory spinal nerves, as the result of non-elimination during the period of gestation, we are, in the treatment of this disease, to address our remedies to the casting off of this poison by the use of drastic cathartics, as the only channel available in the exigencies of the case; and in no case under his observation, when it has been faithfully carried out, has it failed to produce satisfactory results.

This corroborates the views of Dr. Bouchelle, already given in this number.—EDS.

*Perchloride of Iron and Manganese in Necrosis, Fistulous Sinuses and Hydrocele.*—Professor Marcacci, in an essay on this subject in an Italian Medical Publication, arrives at the following conclusions:

1. Perchloride of iron and manganese, injected into fistulous sinuses, destroys the pyogenic membrane, modifies the state of the walls, and favors cicatrization.

2. In necrosis, it acts on the confines of the living bone, stimulating its vessels, so that the detachment and separation of the dead bone are facilitated by the formation of new vessels in the living.

3. In hydrocele, it soon modifies the inner surface of the tunica vaginalis, which becomes filled with plastic exudation, attended with more or less inflammation, according to the quantity and strength of the injection used.

4. It is not necessary that the tunica vaginalis should be distended by the injection; it is sufficient that the liquid be brought into contact with all parts of the membrane.

5. Very little pain is produced by the contact of the solution, but it is not the less efficacious.

6. A weak solution is sufficient, which should be kept in two minutes.

7. In seven cases of hydrocele in which the injection was used, hard œdema followed, but was not a serious complication.—*British Medical Journal, from L'Imparziale.*

CARBOLIC ACID PREPARATIONS.—*Solutton of Carbolic Acid for the Toilette.*—Crystalized carbolic acid, 10 parts; essence of mille-fleur, 1 part; tincture of quillaya saponaria, 50 parts; water, 1000 parts. Mix. The saponine replaces soap with advantage. The above should be employed, diluted with ten times its bulk of water, for disinfecting the skin, for washing the hands after any risk of contagion or inoculation, etc.—*Lemaire.*

*Tincture of Saponine*—As used in the forěgoing preparation, is thus made: Bark of quillaya saponaria, 1 part; alcohol (90°), 4 parts. Heat to ebullition, and filter.—*Le Beuf.*

*Carbolized Water for the Teeth.*—Water, 1000 parts; essence of meat, 2 parts; tincture of saponine, 50 parts; pure carbolic acid, 10 parts. Mix. A dessert-spoonful in a quarter of a tumbler-ful of water, serves as an excellent preparation for cleansing and preserving the teeth.

*Carbolized Ointment.*—Purified lard, 100 parts; carbolic acid, 1 part. Mix. Considered of some service in skin affections; but modified as it is by the fat, it can not replace the aqueous solution of carbolic acid.—*Lemaire.*

*Carbolized Amylaccous Ointment.*—Pure starch 3 parts; hot water, 20 parts. Mix in the ordinary way (the starch being made first into a paste with cold water, and then hot water added), to a stiff consistance; then add olive oil, 1 part; glycerine, 3 parts; carbolic acid, 1 part, and thoroughly mix in a mortar. When cool, this is a soft jelly, which can easily be applied as ordinary ointment. It is much more efficacious than one the base of which is entirely fat, and it is an agreeably cool application.

*Carbolized Oil.*—A. Crystalized carbolic acid, 1 part; boiled linseed oil, 4 parts. Dissolve.—*Lister.*

Pure carbolic acid, 1 part; olive oil, 6 parts. Olive oil is better than linseed oil, as a vehicle, as the latter is more prone to oxi. dation.—*Calvert*

*Carbolized Putty.*—Carbolized oil, about 6 tablespoonfuls; common whiting (chalk), sufficiont to make a firm paste.—*Lister.*

*Antiseptic Lead Plaster.*—Olive oil, 12 parts, (by measure); litharge (finely ground), 12 parts, (by weight); beeswax, 3 parts (by weight); crystalized carbolic acid, 2½ parts (by weight). Heat half the olive oil over a slow fire; then add the litharge gradually, stirring continually until the mass becomes thick, or a little stiff; then add the other half of the oil, stirring as before, till it becomes thick again. Then add the wax gradually till the liquid again thickens. Remove from the fire and add the acid, stirring briskly till thoroughly mixed. Cover up close, and set aside to allow all the residual litharge to settle; then pour off the fluid and spread upon calico to the proper thickness. The plaster made in this way can be spread by machine and kept rolled in stock, and if in a well-fitting tin canister, will retain its virtues for any length of time.—*Lister.*

PRESCRIPTION FOR SOFTENING OF BONES IN CHILDREN:

| | | |
|---|---|---|
| R.—Calcis Phosphatis, | . . . . | dr. ij. |
| Calcis Carbonitis, | . . . . . | dr. i. |
| Sacch. Lactis, | . . . . . | dr. iij.—M. |

S.—10 or 20 grains 2 or 3 times a day in sweetened milk.

*The Sesquichloride of Iron* [*Ferri Chloride, U. S. P.*] *as a Prophylactic in Acute Rheumatism.*—Dr. Anstil (Practitioner) in speaking of the difficulty of managing such cases, says:

"Very different have been the results of treatment since I adopted the use of full doses of sesquichloride of iron from the first moment of such cases presenting themselves. During the past twelve months I have done this fully. Whenever a patient has presented himself with articular pain and slight fever that were plainly of the rheumatic and not of the gouty type, he has been at once placed on thirty or forty minim doses of the tincture of sesquichloride, from three to six of which, according to the severity of the symptoms, have been given in each twenty-four hours. I several times called the attention of students to the fact that (unlike what used to happen) these cases now reappear in my out-patient room on my next hospital day; and in the great majority of instances declare themselves greatly relieved. Since July, 1870, I have treated twenty-nine such patients, of whom thirteen had previously had one or more regular attacks of rheumatic fever, for the symptoms now referred to, with the full doses of iron; and of these, seventeen have lost all pyrexia and spontaneous joint-pain within the three or four days elapsing before my next day at the hospital. Only three have, under my own eyes, developed the full acute disease, and been sent into the ward. Of the remaining nine, four disappeared altogether from my knowledge, so that I cannot say what became of them; the other five, though their symptoms were checked, remained in a state of what might be described as sub-acute rheumatism during from ten to twenty-two days.

"I cannot help remarking with emphasis on the contradiction to old ideas which is involved in the effect of this iron treatment upon the furred tongue. Of course it becomes speedily blacked; but so far from the furring increasing, or the dryness and foul taste becoming more pronounced, what commonly happens is, that after a few days the epithelial coating falls off in considerable patches, and the tongue soon cleans altogether. I believe the prophylactic treatment of rheumatism by the sesquichloride to be one of the most valuable recent improvements in medicine."

*Catechu and Opium as an Astringent in Gleet.*—Dr. R. Locke Johnson extols for the cure of gleet an astringent composed of:

| | |
|---|---|
| Tinct. opii, - - - - | dr. ij |
| Tinct. Catechu, - - - - | dr. ss. |
| Mist. Acaciæ, - - - - | oz. ij.—M. |

To be used twice daily.

He relates one case in which the discharge ceased after the second application, and did not return.—*Medical Press and Circular.*

*Congentital Contraction of the Vagina relieved by Gradual Dilitation by means of Gentian Root.*—Dr. Robert Latour records (La Tribune Medicale, August 13, 1871) two cases of this. The first patient was nearly twenty years of age, and had been married seven years. Their husbands had, neither of them, ever had satisfactory connection with them. Dr. L. introduced into their vaginas a cylinder of root of gentian two millimetres in diameter, and the following day withdrew it, replacing it with another of the size which the first had aquired by imbibition, and this was daily repeated until the vingina was dilated to its normal size. To the cylinders of gentian were attached tapes by which it was attached to the thighs and abdomen to prevent its displacement. Dr. L. directed his patient to preserve entire rest during the treatment. A cure was accomplished in eight days. The first patient became very soon pregnant, and has since given birth to three children without any difficulty. The second patient had not, at the period of the report, yet become pregnant, but coition was effected without any suffering.—*American Journal Med. Sciences.*

*Vaginismus.*—In an interesting article on this affection (American Practitioner, Aug. 1871), Prof. T. Parvin states that undoubtedly the majority of cases of vaginismus can be cured without resorting to the knife. The removal of the cause is frequently sufficient in recent cases. The application of local sedatives may answer in others. Still others may be cured by dilatation, either gradual, as with glass bougies or gum elastic bags distended with air or water, or abrupt, as performed in a manner similar to that originally proposed by Recamier for spasmodic contraction of the sphincter ani.

*Compound Syrup of Assafœtida.*—Mr. J. J. Rambo, of New York, calls attention (American Journal of Pharmacy, September, 1871) to a formula for this preparation, which, he says, he has been for a number of years in the habit of preparing, to obviate the objection felt by most patients to the disagreeable smell and taste of assafœtida, and which has prevented, to a great extent, the more general use of this valuable drug. "The formula I find to answer the purpose effectually, at the same time its medicinal qualities are enhanced by combination with syrup of wild cherry, possesing the valuable therapeutic properties of both :

R.—Infusi pruni Virginianæ, - - Oj.

Assafœtidæ, - . - - oz. j.

Sacch. albi, - - - - oz. xxiv.

Magnes. carb., - - - dr. ij.

Rub the assafœtida and magnesia with the infusion gradually added, so as to make a uniform mixture and filter ; to this, transferred to a bottle, add the sugar and agitate occasionally until it is dissolved. As a result we have a handsome syrup which does not differ in appearance from the syrup of wild cherry.

*Benzoating Ointments Extemporaneously.*—C. F. Bolton (Am. Journal of Pharmacy) recommends the following:

R.—Benzoin pulv. (select.), - - oz. ij.
Ether sulphuric, - - - - oz. iv.
Ol. Ricini, - - - - oz. j.

Introduce the benzoin into a eight ounce bottle, add the ether, macerate for twenty-four hours with frequent agitation, pass through a filter, to the filtrate add ol. ricini, and shake until dissolved ; then transfer to a shallow vessel in order to allow the ether to evaporate spontaneously ; lastly, when the ether has entirely disappeared, place in a wide-mouthed bottle ready for use.

The result from the formula that I have given is of the consistency of a soft extract, one ounce of the extract fully representing an ounce of the benzion in a state that is perfectly miscible with unctuous substances. I benzoated several ointments with this extract in the early part of last April, and allowed them the greater portion of the time to be exposed to the atmosphere, and when I examined them in the fall I could find none of them oxidized in the least, and in the case of ung. hydr. oxidi rubri, the bright orange color was perfectly preserved. I also used it in several prescriptions, and it always gave perfect satisfaction I used it in the proportion of half drachm to the ounce of ointment ; it can also be used very advantageously in preparations for the hair, it being very soluble in alcohol, and perfectly miscible with ol. ricini in combination with alcohol, but insoluble in the fixed and volatile oils in a free state. It is also freely soluble in chloroform.

*Benzoic Acid Ointment for the Relief of Anal Fistula.*—Dr. B. F. Gibbs (Medical Record,) earnestly recommends the use of the following ointment, by inunction, particularly in the incessible blind fistulæ, and remarks that there is an immediate feeling of relief upon its application :

| | | |
|---|---|---|
| R.—Benzoic Acid, | - - - - | grs. xl. |
| Acetat. Morph., | - - - | grs. vj. |
| Simple Cerate, | - - - - | dr. i. |
| Glycerine, | - - - - | q. s. to soften |

as required.

S.—Use every night, by finger of patient, so that the ointment is made to cover the whole ulceration and enter, under pressure, the fistulous opening.

*Antiseptic Cere-Cloth.*—Cloth or thin calico is saturated with cerate (made after the following formula), by simply drawing a portion through it while in a fluid state, or in pieces of any length and width, and rolling, by means of a machine, the calico over cylinders containing cold water, as fast as it has taken up the cerate.

*Strongest Cerate.*—Calvert's pure carbolic acid, liquified, 3 fluid ounces ; olive oil, (colored red with alkanuet root to distinguish the cerate), 1½ fluid ounces ; yellow wax, liquified, 1½ fluid ounces ; paraffin, liquified, 6 fluid ounces. Mix.

*Medium Strength.*—Pure carbolic acid, 2 fluid ounces ; olive oil, 2½ fluid ounces ; yellow wax, 2½ fluid ounces ; paraffin, 5 fluid ounces. Mix.

*Weakest.*—Pure carbolic acid, 1½ fluid oounce : olive oil, 1 fluid ounce and 6 drachms ; white wax, 1 fluid ounce and 6 drachms ; paraffin, 6 fluid ounces. Mix.

*Antiseptic Muslin Gauze.*—Paraffin, 16 parts ; resin, 4 parts ; crystalized carbolic acid, 1 part Melt together. Cheap muslin gauze is dipped in the melted mass and well wrung or pressed while hot. A good substitute for oakum as an antiseptic covering for wounds, unirritating to the most sensitive skin, highly retentive of the acid, and almost destitute of odor. It should, when used, be folded in about 8 layers. It loses the paraffin and resin when washed in boiling water, so the same gauze may be used repeatedly.—*Journal of Pharmacy.*

Cholera Infantum.—Tully's powder (prepared chalk, liquorice powder and pulv. camphor, aa. grs. cc., morphia, grs. x; dose, one or two grains), has been highly recommended by some, but is better suited to cases where the stomach is not very irritable.

*Grey Powder, Soda and Magnesia.*—(Tanner's formula):

| | |
|---|---|
| R.—Hydrarg Cum. Cretæ, . . . | gr. ij, |
| Sodæ Bicarbonitis, . . . . . | gr. ij, |
| Magnesiæ Carbonitis, . . . . | gr. v.—M. |

S.—Make a powder to be taken every other night. An alterative and aperient for children where there is great acidity of the secretions.

Chorea.—Dr. J. Lewis Smith, in a valuable article (Medical Reporter) on chorea, in speaking of various methods of treatment, says, Radcliffe, who has had ample experience in the treatment of nervous affections, writes: "In an ordinary case of chorea the plan of treatment which I have now adopted as a rule for some time, is to give cod-liver oil, in conjunction with hypophosphite of soda, making the draught containing the latter salt the vehicle for the administration of the cod-liver oil." Sometimes camphor or the sesqui-carbonate of ammonia is added. Of more than thirty cases treated in this way, the average duration was under three weeks. Radcliffe began to prescribe these remedies on theoretical grounds, believing that phosphorus and cod-liver oil were required to restore "nerve tone," and the result of this treatment has certainly been such as to commend it to the profession. To children he gives from five to eight grains of the hypophosphite of soda three times daily.

Ergotine as a Hæmostatic.——The Bulletin General Therapeutique quotes from the British Medical Journal a case of Dr. Jamieson, of Berwick, in which the subcutaneous injection of ergotine proved successful in arresting pulmonary hemorrhage on three separate occasions in a man aged forty-one. The Bulletin General, following Dr. Jamieson, ascribes the first idea of the excellent mode of employing ergotine as a hæmostatic to Dr. G. W. Balfour. Three to six grains are sufficient for one injection, and it may be repeated within three minutes if necessary; the action is almost instaneous. A prepared solution may be kept in readiness with a small proportion of spirit or glycerine to preserve it.

*Chilblains.*—Tincture of galls is valuable, applied to unbroken chilblains.

Cholera Infantum.—Dr. Gilbert (Boston Medical and Surgical Journal), in speaking of the treatment of Cholera Infantum, says:

"A remedy new to him was told him by Dr. Dodd, of Prince Edward's Island: after an opiate, equal parts of lime-water and the best salad oil, a teaspoonful every two or three hours. On trying it he was surprised to find it would not only stay on the stomach, but in three cases it also seemed palatable."

*Use of Carbolic Acid to Prevent Pitting After Small-pox.*—Dr. Scott, of Dumfries, writes to the Editor of the Edinburgh Medical Journal, that having experienced the beneficial effects of carbolic acid in preventing disfiguration of the face in severe cases of burning with gunpowder, and with sulphuric acid, he suggested its employment, with this object, in a number of cases of small-pox. It was applied in the following manner. From the first appearance of the eruption, until the completion of desquamation, the face was kept constantly moist with the solution of the acid, in olive oil, (one to eight). The results, he is happy to say, have been most satisfactory; of all the cases treated in the Dumfries Infirmary (several of which were of the confluent type), not one has, on recovery, presented the slightest trace of disfiguration.

*Prescription for Jaundice*—from suppressed action of the liver and in uræmia:

| | |
|---|---|
| R.—Acidi Benzoici, . . . . . . | gr. xx, |
| Glycerini, . . . . . . . | f oz. ss, |
| Aquæ ad, . . . . . . . | f oz. iv.—M |

S.—Dessert-spoonful three or four times a day to a child five years old.—*Tanner.*

General Neuralgia.—M. Gaillard has found that the only remedy capable of combating successfully the general neuralgia of Valleix is the actual cautery lightly applied at a white heat along the course of all the nerves affected. Coupled with a tonic regimen he thus cured two cases—one in eight days, and the other in three weeks, after all other means had failed.—*Medical and Surgical Reporter.*

*Collodion Paint.*—Collodion, one part, and castor oil two parts, is a valuable varnish for various cutaneous affections, excoriations and superficial burns.

*Acute Rheumatism.*—Dr. Wilson Fox (London Lancet), after speaking of the various remedies for the arrest of pain and fev of rheumatism, says:

The fact remains, therefore, that at present the only agent on which reliance can, under the circumstances, be placed, is the external application of cold; and the results of my experience may not be without their value to others. In the first place, it is very distinct from the reports that the surest, most speedy, and effectual method in which it can be applied is by immersion in the bath, while the temperature is observed in the rectum. The patients were simply lifted on a sheet into and out of the bath. After their removal from it they were wrapped in blankets. It is more comfortable to the patients to be immersed in tepid water subsequently cooled, than in cold water. With this precaution the bath is to them the pleasantest of these methods of treatment. Allen C—— repeatedly asked for it while being packed in the wet sheets wrung out of iced water. On several occasions in Allen C——'s case, a rise of temperature was observed to follow immediately after his immersion, and also in the second bath given to Mrs. B——, where a rise of half a degree took place in the first five minutes, but was followed by an immediate fall.

*Hooping Cough Mixture*—(Dr. Fuller's):

| | |
|---|---|
| R.—Zinci Sulphatis, . . . . . . | gr. vij, |
| Extracti Belladonnæ, . . . . | gr. ij, |
| Aquæ, . . . . . . . | f oz. iv.–M. |

S.—Tablespoonful 4 times a day for a child above three years old. Every other day an additional dose may be given. The quantity of belladonna, it is asserted, can be carried to five grains a day, in this manner, without mischief.

*Stimulating Expectorant.*—Useful in pulmonary affections of children, when it is desired to give tartar emetic without producing depression:

| | |
|---|---|
| R.—Vini Antimonial, . . . . . | f dr. i, |
| Spts. Ammo. Aromat,. . . . | f dr. iss,. |
| Syrup Tolutani, . . . . . . | f dr. i, |
| Tr. Camph. Compositæ, . . . . . | f dr. ij,. |
| Aquæ Camphor, . . . . . . | f oz. iss–M. |

S.—One or two teaspoonfuls every third or fourth hour.—*Tanner.*

SUPPURATING SCROFULOUS GLANDS OF THE NECK TREATED BY DRAINAGE, (Middlesex Hospital—London Lancet).—Two patients (children) are attending with scrofula and enlarged cervical glands. In both patients one of the glands had suppurated. Instead of making an incision, Dr. Murry had passed a catgut ligature through the abscess, the contents of which were then allowed to drain away. The glands had quite recovered in one case, and nearly so in the other. No scar was left in either case—a matter of considerable importance, especially in the case of females.

*Chronic Catarrah,*—Attended with debility and restlessness, (Tanner's formula :

| | |
|---|---|
| R.—Syrupi Scillæ, - - - | f dr. ij, |
| Acidi Nitrici diluti, - - | f dr. i, |
| Tr. Hyosciami, - - - | f dr. i, |
| Spts. Chloroformi, - - | f dr. i, |
| Infus. Cinchonæ Flavœ ad. - - | f oz. iv.—M. |

S.—One or two teaspoonfuls two or three times a day, for a child five years old.

*Soothing Ointment.*— R—Liquor Calcis,

| | |
|---|---|
| Olei Amygdal., aa, . . . | f oz. i, |
| Mix well and add fresh lard, . . | oz. i. |

This ointment is soothing and invaluable in irritable ulcers and sores from blister, etc., in children.

*Exhaustive Suppurative Discharges.*—Dr. S. B. Bowsker, (Kansas City Med. Jour.) reports a case of lad dying, apparently, from the exhaustion induced by suppuration from an exostosis of the femur, cured by the following:

| | |
|---|---|
| R.—Ferri Iodidi Syrpuas, - - - | oz. i. |
| Glycerine, - - - - | oz. ij. |
| Ol. Morrhuæ, . - - - | oz. ij.—M. |

S.—Tea-spoonful at meals with Dublin porter and all the food the stomach could bear.

The entire thigh was vesicated with Croton oil.

PLEURO-PNEUMONIA.—Traube gives acid and aqua gummosa, and in very bad cases takes blood from the arm. He finds that wine is very badly borne, and never gives it. His mortality is very small.

